# Variations in the SDN Loop of Class A Beta-Lactamases: A Study of the Molecular Mechanism of BlaC (*Mycobacterium tuberculosis*) to Alter the Stability and Catalytic Activity Towards Antibiotic Resistance of MBIs

**DOI:** 10.3389/fmicb.2021.710291

**Published:** 2021-10-08

**Authors:** Sourya Bhattacharya, Vivek Junghare, Niteesh Kumar Pandey, Subhecchha Baidya, Harsha Agarwal, Neeladrisingha Das, Ayan Banerjee, Debashish Ghosh, Partha Roy, Hirak K. Patra, Saugata Hazra

**Affiliations:** ^1^Department of Biosciences and Bioengineering, Indian Institute of Technology Roorkee, Roorkee, India; ^2^Biochemistry and BIotechnology Area, Material Resource Efficiency Division, CSIR-Indian Institute of Petroleum, Dehradun, India; ^3^Academy of Scientific and Innovative Research, Ghaziabad, India; ^4^Department of Surgical Biotechnology, University College London, London, United Kingdom; ^5^Centre for Nanotechnology, Indian Institute of Technology Roorkee, Roorkee, India

**Keywords:** antimicrobial resistance (AMR), multidrug-resistant (MDR), extensively drug-resistant (XDR), *Mycobacterium tuberculosis*, conserved loop, MD simulation, combinatorial therapeutics, mechanism-based inhibitors (MBI)

## Abstract

The emergence of multidrug-resistant (MDR) and extensively drug-resistant (XDR) tuberculosis calls for an immediate search for novel treatment strategies. Recently, BlaC, the principal beta-lactamase of *Mycobacterium tuberculosis*, was recognized as a potential therapeutic target. BlaC belongs to Ambler class A, which is generally susceptible to the beta-lactamase inhibitors currently used in clinics: tazobactam, sulbactam, and clavulanate. Alterations at Ser130 in conserved SDN loop confer resistance to mechanism-based inhibitors (MBIs) commonly observed in various clinical isolates. The absence of clinical evidence of S130G conversion in *M. tuberculosis* draws our attention to build laboratory mutants of S130G and S130A of BlaC. The study involving steady state, inhibition kinetics, and fluorescence microscopy shows the emergence of resistance against MBIs to the mutants expressing S130G and S130A. To understand the molecular reasoning behind the unavailability of such mutation in real life, we have used circular dichroism (CD) spectroscopy, differential scanning calorimetry (DSC), molecular dynamics (MD) simulation, and stability-based enzyme activity to compare the stability and dynamic behaviors of native and S130G/A mutant form of BlaC. A significant decrease in melting temperature (BlaC T_*M*_ 60°C, S130A T_*M*_ 50°C, and S130G T_*M*_ 45°C), kinetic instability at higher temperature, and comparative dynamic instability correlate the fact that resistance to beta-lactam/beta-lactamase inhibitor combinations will likely not arise from the structural alteration of BlaC, therefore establishing confidence that this therapeutic modality can be potentially applied as a part of a successful treatment regimen against *M. tuberculosis*.

## Introduction

The inquisitor for effective drugs against multidrug-resistant (MDR) and extensively drug-resistant (XDR) *Mycobacterium tuberculosis* introduces different effective combination chemotherapy (Mitchison, 2005). Despite the development of these combination drugs, tuberculosis (TB) remains a significant cause of mortality until the current century, and it is happening in all parts of the world (Global Tuberculosis Report, 2021). One of the primary reasons for the phenomenon is the enzyme beta-lactamase (BlaC), which confers resistance to beta-lactam, the most prescribed antibiotics by clinicians (Kurz and Bonomo, 2012). The *M. tuberculosis* beta-lactamase, BlaC, belongs to Ambler class A (serine-based beta-lactamase), and these are usually susceptible to the beta-lactamase inhibitors or mechanism-based drugs (tazobactam, sulbactam, and clavulanate) currently used in clinics (Drawz and Bonomo, 2010). Interestingly, mechanism-based inhibitors (MBIs) display a unique model of a catalytic mechanism involving two serine residues (Ser_70_ and Ser_130_) instead of one (Ser_70_) for inhibition. In inhibition, a covalent bridge is formed between the primary and secondary catalytic serine (Khanna and Gerriets, 2020). The beauty of the development of this covalent bridge is that it inactivates the enzyme permanently from any further action. This potentiates MBIs toward being one of the most successful inhibitors against all serine-based beta-lactamases and more significantly less prone to the emergence of resistance.

One of the strong possibilities of the emergence of resistance is if the secondary catalytic serine would be mutated to glycine. This is commonly observed in clinical isolates of various organisms like *Escherichia coli*, *Klebsiella pneumoniae*, *Acinetobacter baumannii*, *Proteus*, and *Citrobacter* (Chaibi et al., 1999; Drawz and Bonomo, 2010). Surprisingly, until today, no such clinical isolates (variation of secondary catalytic serine) were isolated from *M. tuberculosis* patient samples. The present report wants to shed light on these surprising findings, which would also assure us toward further long-term use of MBIs in treating TB patients where very few good drugs are available.

The lack of S130G mutation in *M. tuberculosis* in clinical isolates draws our attention to investigating this residue’s role by making laboratory mutants of BlaC (S130G and S130A). The motto and focus of the development of the experimental mutants are to understand the reasons behind not being able to exert this mutation to become resistant against MBIs. In this study, our goal is to investigate the structural and functional features of SDN aka SDG loop present in BlaC beta-lactamase (Lamotte-Brasseur et al., 1991; Atanasov, 2000; Helfand et al., 2003; Thomas et al., 2005). The wild-type BlaC was cloned, and its two variants S130G and S130A were constructed using site-directed mutagenesis strategy. Both the variants and wild-type BlaC were purified. Substrate affinity toward nitrocefin and ampicillin, and inhibition profile of MBIs against wild type and variants were performed.

Further fluorescence microscopy-based LIVE/DEAD cell imaging and growth curve analysis were observed to understand better the effect of beta-lactam and combination of beta-lactam/beta-lactamase inhibitors against clones expressing wild-type BlaC and its variants. *In silico* structural bioinformatics and molecular dynamics (MD) simulation studies were carried out to understand the dynamic nature of the enzyme, flexibility of different loops, and essential residues taking part in catalysis and to maintain enzyme structure. The purified wild-type and variant BlaC were studied through circular dichroism (CD) spectroscopy, differential scanning calorimetry (DSC), and stability-based enzyme activity to determine their melting temperature (T_*M*_) value and optimum temperature of enzyme catalysis. The entire study evaluates the role of serine 130, as both a structural aspect and a functional aspect, by taking interdisciplinary approaches. With the above notions in mind, we sought to determine if substitutions in essential amino acids impair the ability of MBIs to inhibit BlaC and, as a result, jeopardize the use of future beta-lactamase inhibitor combinations against *M. tuberculosis*.

## Materials and Methods

### Sequence Collection, Alignment, and Evolutionary Analysis

The evolutionary prospect of BlaC had been analyzed with two sequence sets. The first set was with class A beta-lactamase to know the relation of BlaC with other related enzymes among the class. The class A sequences were chosen with the in-house protocol. Another set was from different sequences of the beta-lactamase gene of *Mycobacterium* species to observe the position of the query enzyme in it. Initially, the sequence sets were aligned using CLUSTAL W for multiple sequence alignments (Sievers et al., 2011). Then, both analyses were performed using MEGA X (Kumar et al., 2016). The distance tree-based neighbor-joining method was used for evolutionary analysis and Poisson’s correction method for distance calculation (Saitou and Nei, 1987). Further, ambiguous positions were removed for each sequence pair with a pairwise deletion option. The bootstrap method with 1,000 replications was used for the phylogeny test.

The residue conservation was performed for class A beta-lactamase enzymes and the beta-lactamase from *Mycobacterium* species. The sequences were aligned using CLUSTAL W and saved in.fasta format. Then the sequence and modeled structure of BlaC were used as a query sequence and structure, respectively. The ConSurf server was used for calculating the conservation pattern (Ashkenazy et al., 2016). The neighbor-joining method was used to evaluate the evolutionary tree and the Bayesian method for calculating conservation scores (Mayrose, 2004).

### Protein Overexpression and Purification of Wild-Type BlaC and Its Variants

A pET28a-based plasmid carrying a truncated sequence of BlaC (UniProt ID P9WKD3 and GenBank ID: AAB07556.1) was used as the template for site-directed mutagenesis of Ambler position S130 using mutagenic primers (Kurz et al., 2013). Primers used in this study are as follows:

BlaC_S130G-Forward: 5′ GCGATACGCTATGGCGACGGCACCGCC 3′
BlaC_S130G-Reverse: 5′ GGCGGTGCCGTCGCCATAGCGTATCGC 3′
BlaC_S130A-Forward: 5′ GCGATACGCTATGCCGACGGCACCGCC 3′
BlaC_S130A-Reverse: 5′ GGCGGTGCCGTCGGCATAGCGTATCGC 3′

After the mutations were carried out, the plasmid expressing 6 His tagged BlaC and its mutants were transformed into *E. coli* BL21/DE3 cells. Both the wild-type BlaC and its S130G and S130A gene’s nucleotide sequence were justified by automated DNA sequencing (Dr. KPC Life Science Pvt., Ltd., Kolkata, India). Protein expression was induced with 0.5 mM of isopropyl-D-1-thiogalactopyranoside (IPTG) at an optical density at 600 nm (OD600) of 0.6 (Sosa-Peinado et al., 2000). After incubation for 18 h at 16°C, cells were harvested, resuspended in 50 mM of Tris–HCl, containing 250 mM of NaCl, pH 7.2, and disrupted by sonication with a pulse on-time of 5 s and pulse off-time of 10 s in 50 amplitude power. After centrifugation, the soluble extract was loaded onto a Ni-NTA agarose column and eluted with base buffer containing 25 mM of Tris–HCl, containing 300 mM of NaCl, pH 7.5. Further elution was carried out by 30 mM of imidazole and 250 mM of imidazole (Lim et al., 1988).

### Biophysical Characterization of Wild-Type BlaC and Its Variants

#### Circular Dichroism, Thermal Denaturation, and pH Denaturation

All the CD experiments were carried out in a Jasco J-1500 spectrometer with a Peltier-effect temperature controller. Quartz cells with a 0.1-cm path length were used for all experiments. For thermal denaturation, 2 mg/ml of wild-type BlaC, S130G, and S130A variant were monitored for helical content by CD at λ222 between 25 and 90°C with a heating rate of 5°C/min (Christov et al., 2001; Bhattacharya et al., 2020). The same experiment was performed against the increasing concentration of pH. BlaC, S130G, and S130A mutants were incubated in the buffer of different pH values before experiments. Each buffer of a different pH was run as blank. Respective ellipticity at 222 nm was plotted against increasing pH to observe the folding unfolding transition of BlaC, S130A, and S130G under the influence of pH.

#### Differential Scanning Calorimetry

The differential calorimetry process is ideal for the thermal analysis of a macromolecule. All the DSC experiments were carried out in a MicroCal-VP-DSC (GE Healthcare, Chicago, IL, United States). The sample cell and the reference cell in the DSC system’s thermal core were both heated. Firstly, baseline equilibration was carried out by degassed appropriate buffer. During DSC measurement, the reference cell was filled with buffer, and the sample cell was filled with 80 μM of purified wild-type BlaC, and its S130G and S130A variants were heated at a constant scan rate. The temperature difference between the cells across the thermal gradient directly gives us the thermal denaturation point of both the wild-type and mutant proteins of BlaC protein (Durowoju et al., 2017).

### Biochemical Characterization of the Enzyme

#### Kinetic Profiling and Temperature Rate Profile of BlaC and Its Variant

Kinetic profiles, including the hydrolysis parameter of different beta-lactams toward BlaC, and its variants were collected on a Cary 60 UV-Vis spectrophotometer with 16 cuvette holder Peltier water bath (Agilent Technologies, Santa Clara, CA, United States). All kinetic reactions were performed in 0.1 M of phosphate buffer of pH 7.2 at 25°C (Hazra et al., 2015). Respective substrates were prepared freshly in the same buffer. Reactions were started by taking the range of substrate concentration and ultimately adding enzyme in a final concentration of 10 nM in the reaction mixture to start the reaction (Hazra et al., 2014). The initial velocities were calculated from the linear phase of the reaction curve in a particular time frame and plotted against the substrate concentration to determine various kinetic parameters like K_*m*_ and k_*cat*_ (turnover number) of the enzyme toward different substrates (Sabini et al., 2006). The potency of inhibitors toward BlaC and its variants were measured with inhibition kinetics, where a concentration gradient of the inhibitors was used against the reference substrates nitrocefin (Sabini et al., 2007). The rate profiling at various temperatures was carried out at temperatures ranging from 20 to 60°C by taking nitrocefin as a standard substrate (Bhattacharya et al., 2020). A 100 mM phosphate buffer of pH 7.2 was taken for all the experiments. The final concentration of wild-type BlaC and S130G variants was taken as 10 nM. At different temperatures, kinetic parameters were calculated for both the proteins (Bhattacharya et al., 2020).

### Interaction Study of BlaC and Its Variants

#### Isothermal Titration Calorimetry

Thermodynamic parameters and binding affinity of BlaC and its variants with MBI sulbactam were assessed using isothermal titration calorimetry (ITC). All the experiments were carried out using MicroCal iTC200 Systems (GE Healthcare, United States). Both inhibitor and protein (protein and ligand) were dissolved in the same 0.1 M phosphate buffer of pH 7.2. A control reaction was designed by taking buffer in cell and injection for understanding buffer–buffer interaction (Verma et al., 2011). Isothermal interactions between BlaC and its variants with sulbactam were measured by titrating over 16 injections using 60 μl of sulbactam (100–500 μM) and proteins in a sample cell with a concentration of 50 μM of 200 μl (Mandal et al., 2014). All the proteins and ligands were degassed before the respective experiment.

#### Homology Modeling and Docking

The BlaC from *M. tuberculosis* amino acid sequence was taken from the UniProt database having ID P9WKD3. Then the sequence was used to perform BLAST using the Protein Data Bank (PDB) databank as a search database. It was achieved by using the SWISS-MODEL online web server. The modeled structures were used as an input to perform energy minimization through GROMACS v2018.1 (Van Der Spoel et al., 2005). The AMBER-ff99sb-ILDN force field (Lindorff-Larsen et al., 2010) and tip3p (Price and Brooks, 2004) were chosen as force-field and water models for this calculation. The solvated 1-nm box was neutralized by adding ions. Energy minimization was carried out using the steepest descent algorithm (Fliege and Svaiter, 2000) and the conjugate gradient algorithm (Ponder and Richards, 1987), with a converge force of less than 1,000.0 kJ/mol/nm and a cumulative step size of 50,000. The energy-minimized structures were refined and visualized using COOT and PyMOL.

For molecular docking, the small-molecule structure generation was carried out using ChemDraw 2D and Chem3D for two-dimensional and three-dimensional models (Cousins, 2011). The three-dimensional model was energy minimized using MM2 (Broeker et al., 1991) minimize tool of Chem3D. Both small-molecule and enzyme structures were converted to pdbqt using Auto Dock Tools (Auto Dock Tool, 2016). The knowledge-based grid generation was performed for the enzyme. The structure with PDB ID 6NVU (Cifuentes-Castro et al., 2020) was used as a template for docking. The grid was centered at (34.78, 44.37, and 44.06) with dimension 20 on each axis. After the configuration file was prepared, molecular docking was performed using AutoDock Vina (Trott and Olson, 2009) for 10 runs. The pose selection was performed by aligning with the template structure used for docking. Then, the final selected structures were used for visualization using PyMOL.

### Dynamic Characterization of BlaC and Its Variants

The crystal structures of two beta-lactamase proteins from *E. coli* (PDB ID: 1XPB) (Fonzé et al., 1995) and *M. tuberculosis* (PDB ID: 2GDN) (Wang et al., 2006) were obtained from RCSB PDB^1^ and used as starting models for the MD simulations. Crystal structures of TEM1 from *E. coli* (1XPB) and BlaC from *M. tuberculosis* (2GDN) have resolutions of 1.90 and 1.70 Å, respectively. The crystal structures of the BlaC wild types were used as templates for homology modeling of different variants. The modeled structures are validated using PDBsum.

**Table:** 

Sl. No.	System description
1	Ec_Wt
2	Mt_Wt
3	Mt_S130G
4	Ec_S130G

**Table:** 

Sl. No.	System description	Organism
1	1XPB	*E. coli*
2	2GDN	*M. tuberculosis*

All MD simulations were performed using the GROMACS 5.0.2 (Hess et al., 2008) and OPLS-AA (Huang et al., 2012; Pronk et al., 2013) all-atom force field implemented on Intel Xeon Quad-Core W3530 2.8 8M 1366 Processor with LINUX environment. The entire BlaC mutant and the wild-type BlaC were solvated in a cubic box of the SPC216 water molecule (Miyamoto and Kollman, 1992). Water molecules were replaced by Na^+^ to neutralize the net charge of the system for BlaC protein. All protein atoms were maintained at a distance equal to 1.0 nm from the box edges. The solvated systems were subjected to energy minimization with 3,000 steps by the steepest descent minimization method. After energy minimization was performed, all the minimized systems were equilibrated for 100 ps by position restrained MD simulation to maintain pressure and temperature of systems and to relax the solvent. Following equilibration, all systems were then subjected to final MD simulations for 100 ns each at 300 K. Periodic boundary conditions were applied under isothermal and isobaric conditions using the Berendsen coupling algorithm with a relaxation time of 0.1 and 0.2 ps, respectively, (Weber et al., 2000). The LINCS algorithm was used to constrain bond lengths using a time step of 2 fs for all the systems (Hess et al., 1997). Electrostatic interactions were calculated using the particle mesh Ewald method and van der Waals, and the columbic interactions were calculated with a cutoff at 1.0 nm (Darden et al., 1999). The tools provided by the GROMACS program package were utilized to analyze different MD trajectories. PyMOL and Xmgrace programs were used to analyze and to prepare publication-quality figure.

### Network Analysis

The S_130_DN_132_ loop was observed in class A beta-lactamase and is a conserved motif for this class. As BlaC from *M. tuberculosis* has G_132_ in place of N_132_, the residue network was analyzed for the different mutations performed. The network was prepared based on the interaction of enzyme residues making polar contact with the loop residues. All the residues around the 4 Å from the loop residues were analyzed for searching for a contact. Then, the distances were evaluated, and the residues and atoms were noted. The network study was performed using the environmental distance tool of COOT, and PyMOL was used to represent it.

### AO/EB Double Staining for Fluorescence Microscopy

The fluorescent dye acridine orange (10 mg) and ethidium bromide (10 mg) were dissolved in 10 ml of phosphate-buffered saline (PBS). Then a bacterial suspension of 1.0 × 10^8^ bacteria expressing wild-type BlaC, S130G, and S130A mutations were harvested by centrifugation (5,000 × *g* rpm for 10 min). After being harvested, the cell pellets were resuspended in PBS and mixed thoroughly before being harvested again, and the pellets were then resuspended in PBS. Beta-lactam and beta-lactam/beta-lactamase inhibitor combinations were added according to Clinical and Laboratory Standards Institute (CLSI) guidelines into the bacterial suspension expressing wild-type and mutant BlaC. All the treated samples were incubated at 37°C for 6 h. Then 100 μl of dye was added and incubated for 15 min in ice. After incubation, all the samples were harvested, and the cell pellets were resuspended in PBS. Then, 10 μl of each sample was placed on a clear glass slide with a glass coverslip and observed under a fluorescent microscope (EVOS FLoid; Invitrogen, Carlsbad, CA, United States).

### Growth Curve Analysis

Growth inhibition of clones expressing wild-type BlaC, S130G BlaC, and S130A BlaC were inoculated into Luria-Bertani (LB) broth, grown at 37°C to an OD600 of 1.0 diluted to a starting OD600 of 0.18. Fractions were inoculated in duplicate with the following compounds at a concentration of 10 μg/ml of ampicillin and 10 /10 μg/ml of ampicillin/clavulanate. Growth was assessed 24 h by measuring OD600 and compared with that of control culture.

## Results

### Evolutionary Relatedness of BlaC, SDN Loop Conservation, and Rational Basis of Mutation Study

**FIGURE 1:**
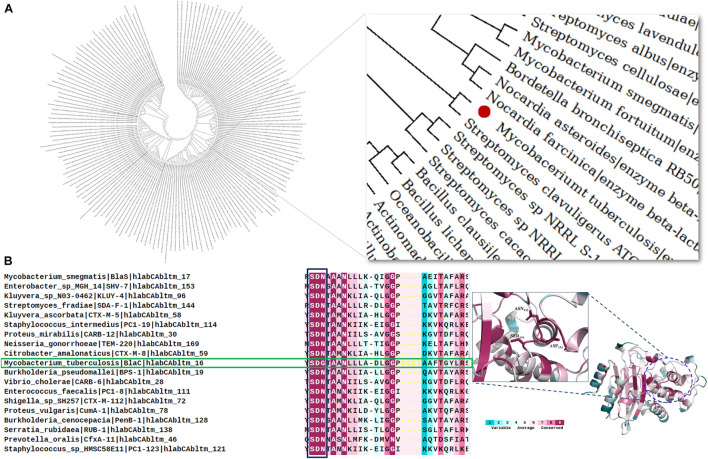
Evolutionary and conservation analyses of BlaC beta-lactamase. **(A)** Relatedness of BlaC beta-lactamase from *Mycobacterium tuberculosis* (highlighted in the red sphere) with class A beta-lactamase from other species. **(B)** Conservation analysis of SDN loop in different class A beta-lactamases. *M. tuberculosis* (green box) has an SDG loop instead of an SDN loop.

The evolutionary analysis of BlaC beta-lactamase was performed here in two aspects. Firstly, we showed the relatedness of BlaC among all the other class A beta-lactamase from different species. Among the 160 selected class A sequences taken for the phylogenetic analysis, in the evolutionary map in Figure 1A, BlaC from *M. tuberculosis* is found to be similar to beta-lactamase from *Streptomyces clavuligerus*. BlaC showed significant relatedness with two *Nocardia* species, *Nocardia farcinica*, and *Nocardia asteroides* (acid fast). From the phylogenetic analysis in Figure 1A, BlaC has a distant similarity with *Bacillus clausii* and *Bacillus licheniformis*. Secondly, to understand the evolutionary analysis of BlaC among beta-lactamase from different *Mycobacterium* species, we found that BlaC showed direct similarity with class A beta-lactamase from *Mycobacterium bovis*, *Mycobacterium canettii*, *Mycobacterium orygis*, and *Mycobacterium lactis* (Supplementary Figure 1). Conservation analysis provides us a pictorial description of the conservation of critical residue and loop. The conservation analysis in Figure 1B shows that the SDN loop is conserved for all the class A beta-lactamase as per the color code. Instead of the SDN loop in all the other members, we found that *M. tuberculosis* has an SDG loop highlighted with a green box. We also found the conservation of the SDN loop in the structure of beta-lactamase. This concludes the importance of this SDN loop in catalysis and is a rational choice for our further study of mutagenesis of this loop for the sequence requirement against MBIs.

### Production of Native, S130G, and S130A Variants of BlaC

**FIGURE 2:**
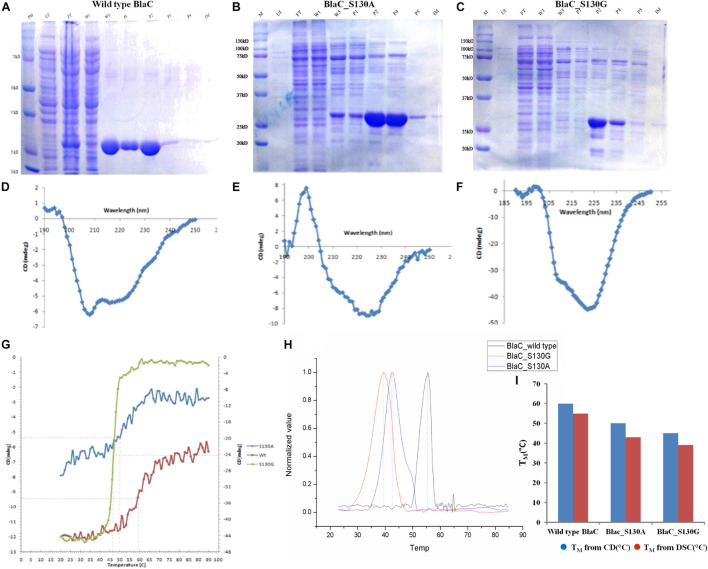
Production and biophysical characterization of wild-type and variant BlaC. **(A)** Production of wild-type BlaC. **(B)** Production of S130A BlaC. **(C)** Production of S130G BlaC. **(D)** CD spectra of wild-type BlaC. **(E)** CD spectra of S130A BlaC. **(F)** CD spectra of S130G BlaC. **(G)** Thermal denaturation comparison of wild-type and variant BlaC by CD spectroscopy. **(H)** Determination of melting temperature using DSC. **(I)** Comparison of melting temperature of wild-type BlaC and its variant coming from CD spectroscopy and DSC. CD, circular dichroism; DSC, differential scanning calorimetry.

We have purified both wild type and its variants to homogeneity as explained in the “*Materials and Methods”* section. The sodium dodecyl sulfate–polyacrylamide gel electrophoresis (SDS-PAGE) analysis revealed single bands of similar sizes for all variant proteins (Figures 2A–C). We have quantitatively measured the respective yield of BlaC and its variants by the Bradford quantitative assay. We found the production of wild-type BlaC is higher than its variants from equal culture volume taken.

### Biophysical Characterization to Understand the Alternation in Stability With the Incorporation of Single Amino Acid Mutation

The main significance of performing CD with the wild type and mutants of BlaC is to understand the influence of secondary structure changes associated with the incorporation of designed mutation. CD spectra analysis of wild-type BlaC and its two variants, S130A and S130G, showed different structural contents when monitoring a wavelength scan from 190 to 260 nm (Figures 2D–F). There is a striking decrease in T_*M*_ observed from wild type to mutant protein, while thermal denaturation was monitored with 2 mg/ml of wild-type BlaC and the S130G and S130A variants with helical content by CD at λ222 nm between 25 and 90°C with a heating rate of 5°C/min (Figure 2G). The melting temperature of wild-type BlaC was 60°C, the melting temperature S130A variant was 50°C, and the melting temperature of S130G was found to be 45°C. The single amino acid mutation in BlaC protein makes the protein more unstable, suggesting the role of this amino acid in maintaining the structure of the protein and function with a net decrease of 15°C of the T_*M*_. We further determine the stability of wild-type BlaC and its variants toward variable pH. Respective native and mutants BlaC were incubated in variable pH (pH 3 to pH 11) and further monitored a wavelength scan in the far UV region (190 to 260 nm). Ellipticity at 222 nm for each of the spectra was plotted against variable pH to measure the change in ellipticity (better to understand the alteration in the secondary structure of the protein) by the influence of pH. Supplementary Figures 2–4 show pH denaturation of native, S130G, and S130A BlaC under the influence of pH. We observed a significant loss of helicity at lower and higher pH values. All the tested proteins showed higher helical content at the neutral pH in the range of pH 6.5 to pH 7.5.

Differential scanning calorimetry is the simplest way to determine the stability of proteins in their natural three-dimensional configurations. We calculate the heat capacity (ΔCp) of protein (wild-type BlaC, BlaC_S130A, and BlaC_S130G) denaturation under the influence of heat using the DSC method. Because of the absorption of heat by the various covalent and non-covalent bonds associated with the protein, the data obtained from the DSC curve provided us with information on protein stability during thermal denaturation. All the associated DSC curves of wild-type BlaC and its two variants are listed in Supplementary Figure 5. We have found that the melting temperature of wild-type BlaC is 54.62 ± 0.025 higher than S130A of 43.13 ± 0.023 and S130G of ±0.027. Like the melting temperature we obtained from CD, we observed a significant decrease in melting temperature when the serine residue mutates to alanine and glycine in DSC (Figure 2I). The respective ΔCp values are normalized and represented in Figure 2H to compare the melting temperature of wild-type BlaC and variants.

### Biochemical Characterization of the Enzyme

#### Functional Role of S130 in Substrate Hydrolysis and Emergence of Inhibitor Resistance: Temperature-Dependent Rate Profile of BlaC and Its Variant to Observe the Alternation in Catalytic Activity

We selected nitrocefin (chromogenic cephalosporin) and ampicillin (penicillin) as substrate for wild-type BlaC and its S130G mutants for steady-state kinetics. From the kinetic parameter listed in Table 1, we found that the hydrolysis efficiency of S130G toward nitrocefin and ampicillin is lower than that of wild-type BlaC. Also, Km values of both the substrates were higher toward S130G than wild-type BlaC, which suggests lower substrate affinity. The higher Km and decrease in catalytic efficiency of S130G may be due to the absence of critical interaction performed by serine 130 toward these substrates. We have measured the inhibitory potential of MBIs against wild-type BlaC and S130G. The k_*i*_ value of three inhibitors toward wild-type BlaC is lower than the S130G variant. All the inhibition kinetic data are listed in Table 2. The overall increase in k_*i*_ against three MBIs toward S130G variants suggests that the laboratory mutants acquire the resistance. This implicates the functional role of Serine 130 toward different MBIs. We further calculated hydrolysis of wild-type BlaC and S130G variant toward nitrocefin at variable temperatures. The result tabulated in Table 3 shows that wild-type BlaC has optimum catalysis temperature approximately 40 to 50°C, whereas S130G has an optimum temperature of fewer than 40°C. This observation correlates with the melting temperature that we observed in CD spectroscopy. The optimum temperature for catalysis strikingly decreases in the experimental mutant S130G, suggesting serine 130 in maintaining the enzyme structure.

**TABLE 1:** Steady-state kinetic parameters of wild-type BlaC and its variant S130G toward nitrocefin and ampicillin.

	K_*m*_ (μ M)	K_*cat*_ (min^–1^)	K_*cat*_/K_*m*_ (μ M^–1^ min^–1^)
Nitrocefin+WT BlaC	72 ± 4	82 ± 2	1.13
Nitrocefin+S130G	230 ± 8	8.5 ± 1.5	0.036
Ampicillin+WT BlaC	65 ± 2	26 ± 2.5	0.4
Ampicillin+S130G	286 ± 11.2	25 ± 9	0.087

**TABLE 2:** Inhibition kinetic parameter of three mechanism-based inhibitors (clavulanate, sulbactam, and tazobactam) toward wild-type BlaC and S130G variant.

	K_*i*_ (μ M) (clavulanate)	K_*i*_ (μ M) (sulbactam)	K_*i*_ (μ M) (tazobactam)
WT BlaC	26 ± 1.5	16 ± 1	52 ± 3
S130G	100 ± 5.1	111 ± 2.3	154 ± 12.6

**TABLE 3:** Temperature-dependent rate profiling of BlaC and its variant S130G beta-lactamase toward nitrocefin at variable temperatures.

Temperature	Protein name	K_*m*_ (μ M)	V_*max*_ (μ M/min)	K_*cat*_ (min^–1^)	K_*cat*_/K_*m*_ (min^–1^ μ M^–1^)	
20°C	**WT BlaC**	**79 ± 13.45**	**0.62 ± 0.23**	**31 ± 0.51**	**0.39**	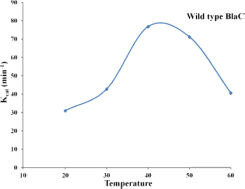
	S130G	245.04 ± 13.45	0.092 ± 0.03	4.6 ± 0.45	0.018		
30°C	**WT BlaC**	**76 ± 4.3**	**0.84 ± 0.3**	**42.8 ± 1.02**	**0.56**		
	S130G	214 ± 7.8	0.165 ± 0.03	8.25 ± 1.5	0.038		
40°C	**WT BlaC**	**78 ± 2.1**	**1.52 ± 0.21**	**76.83 ± 11.2**	**0.97**		
	S130G	233 ± 9.10	0.207 ± 0.07	10.38 ± 2.5	0.044		
50°C	**WT BlaC**	**109.16 ± 16.10**	**1.42 ± 0.02**	**71.25 ± 5.65**	**0.65**		
	S130G	107.34 ± 16.10	0.0025 ± 0.001	0.125 ± 0.05	0.001		
60°C	**WT BlaC**	**151.6 ± 18.3**	**0.81 ± 0.12**	**40.65 ± 13.21**	**0.26**	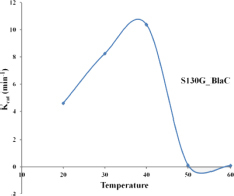
	S130G	314.36 ± 98.19	0.0019 ± 0.001	0.095 ± 0.03	0.0003		

### Interaction of Mechanism-Based Inhibitor With the Enzyme to Observe the Role of S130 in Substrate Recognition

We performed ITC with sulbactam, an MBI with wild-type BlaC, S130A, and S130G mutants to understand the stoichiometry and binding affinity of beta-lactamase and beta-lactamase inhibitors. ITC thermogram in Supplementary Figure 6 correlates the affinity of sulbactam with three tested proteins. All the thermodynamic parameters were tabulated in Supplementary Figure 5. We have observed that the binding affinity of sulbactam is higher against wild-type BlaC than the S130A and least observed in S130G (Supplementary Table 1). The resulting decrease in binding affinity in the S130 variant may be coming from the lack of interaction of serine 130 with the substrates.

With the help of molecular docking, we have inspected the binding of clavulanate (a beta-lactamase inhibitor) with wild-type BlaC and S130G mutant. According to the molecular docking data, the S130G mutation in BLAC’S SDG loop has a major effect on clavulanate binding. The binding affinity of clavulanate toward wild-type BlaC (−6.5 kcal/mol) is relatively effective as compared with that of S130G (−4.8 kcal/mol). The relative decrease in binding energy suggests that clavulanate could not bind S130G mutants compared with wild-type BlaC. Apart from the binding energy, we also have found possible critical interaction between ligand molecule and essential residues within wild-type BlaC and S130G mutants. All the interatomic distances, as well as interacting residues, were tabulated in Supplementary Table 2. The table found that wild-type BlaC binds with clavulanate by hydrogen bonds with various vital residues like Ser_84_, Ser_142_, Tyr_141_, Thr_232_, Arg_236_, and Thr_253_. The S130G mutants bind with clavulanate, resulting in a weak hydrogen bonding network comprising Ser_84_, Thr_232_, Thr_251_, Lys_250_, and Arg_236_. A significant lower binding energy and hydrogen bond interaction were observed in molecular docking when clavulanate binds with S130G mutant in comparison with WT BlaC.

### Dynamic Instability of SDG Loop Harboring BlaC in Comparison With SDN Harboring TEM and Its Corresponding S130 Mutation

**FIGURE 3:**
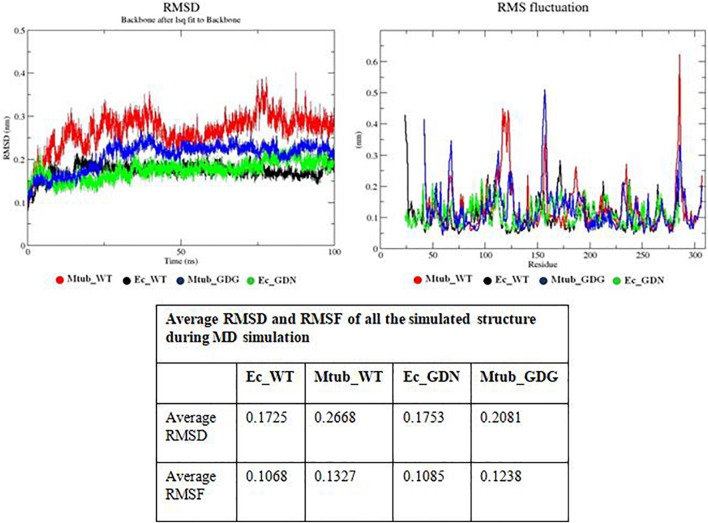
Molecular dynamics simulation finding. The top panel describes RMSD analysis of wild-type BlaC, wild-type TEM, S130G mutant of BlaC, and S130G mutant of TEM. All the respective systems were designated with a particular color in the figure. The bottom panel describes the RMSF analysis of all the above systems. The bottom table indicates the average RMSD and RMSF values of all the simulated systems. RMSD, root mean square deviation; RMSF, root mean square fluctuation.

Structures of the wild-type protein and its mutant proteins can be best compared by using root mean square deviation (RMSD). In the course of the simulation, it measures the difference between backbone atoms’ positions of the protein relative to their starting structures. The smaller the deviation, the more spatially equivalent the two states (starting and simulated) of the proteins are, and the more stable the protein structure is. Here, we have calculated the RMSD of four systems as wild-type BlaC and its S130G variant and wild-type TEM from *E. coli* and its S130G variant. All the RMSD values were calculated regarding the energy-minimized structures of wild-type BlaC and its S130G variant, and wild-type TEM and its S130G variant. From the average RMSD value that we obtained from the MD simulation trajectory in the table of Figure 3, we found that the average RMSD value of wild-type BlaC (0.2668 nm) is much higher than that of wild-type TEM (0.1725 nm). These observations conclude the relative instability of the wild-type BlaC in comparison with wild-type TEM. Figure 3 shows the higher deviation of simulated structure wild-type BlaC in red color than wild-type TEM in black color. The S130G mutation of BlaC has an average RMSD value of 0.2081 nm, and the S130G mutation of TEM has an average RMSD of 0.1753 nm calculated from the MD simulation trajectory. In Figure 3, we have seen that the modulated structure of the GDG mutant of BlaC in blue has a characteristic higher deviation than modulated structure GDN mutant of TEM. These findings suggest that WT BlaC has a higher deviation than wild-type TEM that confer less stability of the BlaC compared with TEM. Again, serine-to-glycine mutation of BlaC confers more structural instability than the serine-to-glycine mutation in TEM beta-lactamase.

A more detailed picture of differences in residue mobility within and between simulations can be obtained from graphs of the root mean square fluctuation (RMSF) of Cα atoms relative to the average structure. Such fluctuations have been calculated for their simulations. Analysis of RMSF values clearly shows the structural flexibility of the loop region compared with the rest of the protein structure. It also shows the variation of different residues that directly correlate with catalysis. Here, we calculated the average RMSF for wild-type BlaC and its S130G mutation, and wild-type TEM and its S130G mutation to understand the overall residual flexibility of each modulated structure. The table in Figure 3 shows that the average RMSF value for wild-type BlaC (0.1327 nm) is greater than the average RMSF value for wild-type TEM (0.1068 nm). This suggests that the overall residual flexibility is more significant than TEM. On the other hand, the GDN mutant of TEM also has lower residual flexibility (0.1085 nm) than the GDG mutant of BlaC (0.1238 nm).

### Intramolecular Residue Interaction Network of SDG Loop in Comparison of SDN Loop

**FIGURE 4:**
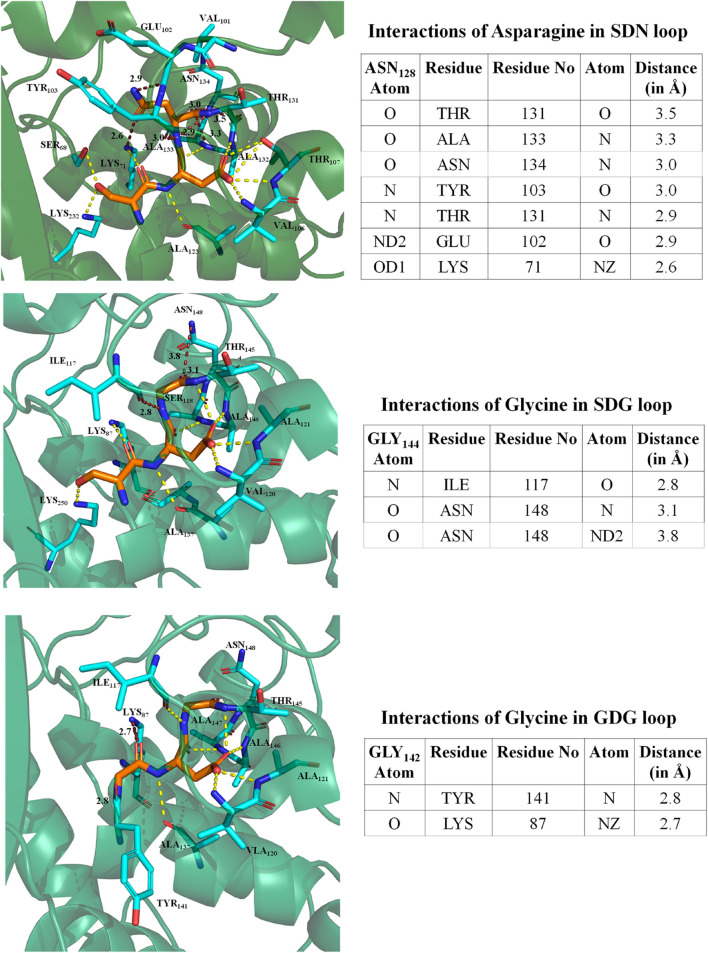
Intramolecular residual interaction network analysis. The top panel describes the residual interaction network of asparagine in the conserve SDN loop of TEM beta-lactamase. The middle panel describes a possible network of glycine in the SDG loop of BlaC beta-lactamase. The bottom panel shows an intramolecular network of GDG mutant in BlaC beta-lactamase.

We have performed an intramolecular residue interaction network of conserved S_130_DN_132_ loop in class A beta-lactamase. As BlaC from *M. tuberculosis* has G_132_ in place of N_132_, the residue network was analyzed for the different mutations performed and TEM to understand the role of intraresidual interaction in enzyme stability and catalysis. The analysis found that N_132_ in TEM interacts with primary catalytic residues like K_73_, E_166_, and S_70_. It interacts with other residues like T_134_, A_136_, N_137_, Y_106_, and E_105_. We found that G_132_ in the SDG loop has interaction only with residues I_117_ and N_137_. In the S130G mutation, the interaction of G_130_ has even less interaction with only Y_129_ and K_73_. All the intramolecular interactions are depicted in Figure 4.

### Morphological Observation of (Clone Expressing BlaC and Its Variant) Beta-Lactam and Beta-Lactam/Beta-Lactamase Inhibitor-Treated Bacteria

**FIGURE 5:**
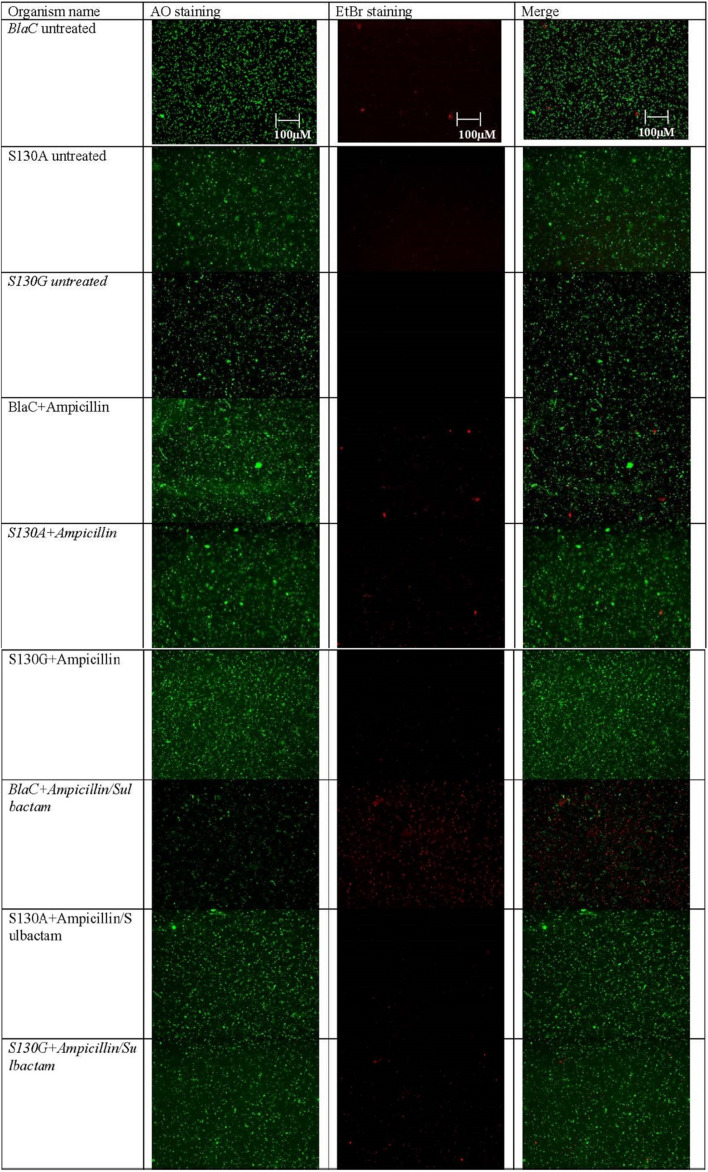
Representative fluorescence microscopy images of AO/EB-based dual staining of clone expressing wild-type BlaC and its two variants S130G and S130A after incubation with ampicillin, sulbactam, and ampicillin/sulbactam to determine the emergence of resistance occurring through particular point mutation.

Clone expressing wild-type BlaC and its S130G and S130A mutations were selected for LIVE/DEAD bacterial viability assay after treating them with ampicillin, ampicillin/sulbactam, and sulbactam using a combination of AO/EB as fluorescent dyes. Intact bacterial cells were stained green, and the cell with compromised cell membranes due to drug treatment was stained as red due to permeation of EB through the cell membrane and their subsequent intercalation with DNA. As shown in Figure 5, bacteria expressing both the wild-type and mutant BlaC gene remained intact when treated with ampicillin. Our data showed that the number of dead cells was more when wild-type BlaC was treated with ampicillin/sulbactam than with the two mutants. These results implicate resistance shown by two experimental mutants S130G and S130A against sulbactam. However, the gross impression of the impact of two mutations in the SDN loop of BlaC was found to inhibit against most commonly used beta-lactamase inhibitors.

### Influence of Growth Rate in the Presence of Beta-Lactam and Beta-Lactam/Beta-Lactamase Inhibitor Combination

**FIGURE 6:**
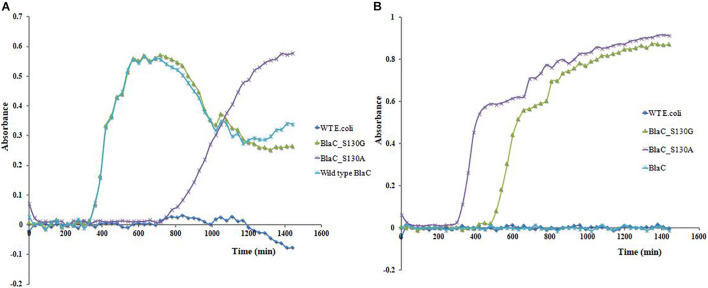
Clone’s growth curve analysis expressing wild-type BlaC, BlaC_S130A, BlaC-S130G, and wild-type *Escherichia coli*. **(A)** Growth curve analysis in the presence of 10 μg/ml of ampicillin. **(B)** Growth curve analysis in the presence of 10 μg/ml of ampicillin and 10 μg/ml of sulbactam (beta-lactam/beta-lactamase inhibitor combination).

Clone expressing wild-type BlaC, BlaC_S130A, BlaC_S130G, and wild-type *E. coli* (no beta-lactamase) was subjected for growth curve analysis under the presence of ampicillin and ampicillin/sulbactam. When all the organisms are treated with ampicillin (Figure 6A), we observed the growth of wild-type BlaC and both variants. Wild-type *E. coli* cannot grow in ampicillin, suggesting ampicillin resistance shown by all the native and variant beta-lactamases. When treated with ampicillin/sulbactam combination, the growth of wild-type *E. coli* and wild-type BlaC stopped because of beta-lactamase inhibitor, which inhibits the native BlaC. S130G and S130A variants of BlaC remain susceptible in the presence of beta-lactamase inhibitor due to the emergence of beta-lactamase inhibitor resistance (Figure 6B).

## Discussion

**FIGURE 7:**
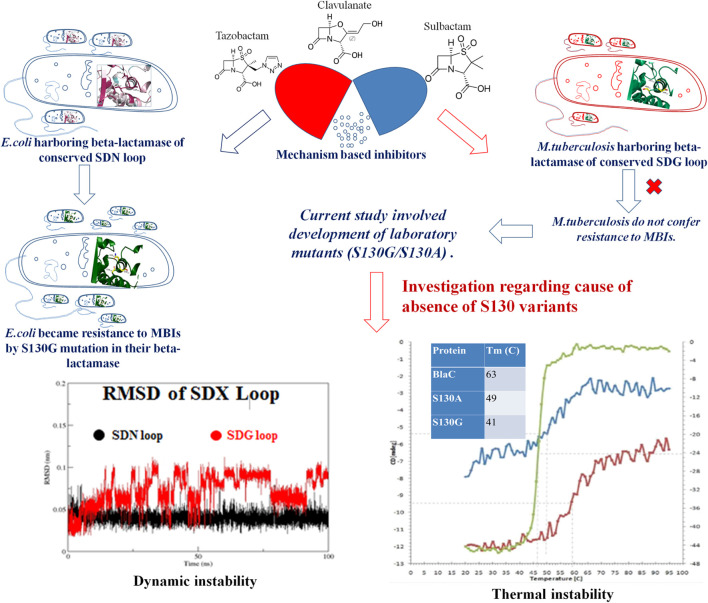
A conclusion model to describe the problem statement and outcomes from the study.

The primary goal of our study was to determine if substitutions that are expected to confer resistance to MBIs have a significant effect on *M. tuberculosis* susceptibility to MBIs. To test this, we have aligned class A beta-lactamase from the different organisms and found that the SDN loop is conserved. *M. tuberculosis* has an SDG loop instead of a conserved SDN loop in all the other beta-lactamases of class A. We have found clinical isolates of various organisms harboring S130G mutation to develop resistance against MBIs (Chaibi et al., 1999; Drawz and Bonomo, 2010). Fortunately, there was no evidence of S130G variants in *M. tuberculosis*. The uniqueness of the unavailability of S130G mutants/isolates was reported by few groups (Kurz et al., 2013). Still, no one commented regarding the molecular reason behind this absence of that clinical isolates. That is the novelty of current work, where we have constructed two variants using the technique of site-directed mutagenesis targeting the S130 position (S130G and S130A). We compared the effect of designed variants with respect to wild-type BlaC using various interdisciplinary approaches and characterize them biophysically and biochemically (Figure 7). We tested their effect of bringing resistance toward MBIs. We have found lower substrate specificity and beta-lactam hydrolysis of S130G in comparison with WT BlaC. This result correlates with the previous result suggesting the importance of serine in the SDN loop (Ser130) as the base catalyst in substrate recognition and beta-lactam ring opening. Inhibition kinetics with various inhibitors against WT BlaC and S130G clearly showed the emergence of inhibitor resistance-conferring by S130G mutation. Detailed binding potential of beta-lactamase inhibitors with WT BlaC and S130G using molecular docking and ITC indicated lower binding affinity of beta-lactamase inhibitor in the binding site of S130G mutant. To answer why *M. tuberculosis* cannot produce S130-related clinical variants, we performed various comparative studies using dynamic, biochemical, and biophysical approaches. MD simulation study has revealed an apparent comparative enhancement in flexibility of the SDG loop of BlaC compared with the SDN loop of other class A beta-lactamase taking TEM (*E. coli*) as a representative.

Further, the S130G variant of BlaC showed higher dynamic instability than the S130G mutants of TEM from *E. coli*. In various *in vitro* experiments, we compared wild-type BlaC and its variants by successfully purifying the enzyme. We performed a thermal denaturation study of wild-type BlaC, S130A, and S130G variant of BlaC with CD spectroscopy and DSC. We found notable differences in the melting temperature in the case of S130G. The experimental mutant S130G has a melting temperature of 15°C lower than its wild-type partner. From the analysis, we inferred that a single mutation in BlaC makes the enzyme structurally unstable. To understand the effect of the mutation biochemically, we performed temperature-dependent rate profiling of both the wild-type BlaC and its S130G variant. We found that S130G mutants showed the catalysis only up to 30°C and had an optimum temperature of between 30 and 40°C. Wild-type BlaC showed catalysis even up to 50°C. However, we found that our experimental mutant S130G showed resistance to MBIs with a higher k_*i*_ value. These detailed studies showed the mutants to bring resistance and showed the molecular reasoning behind the unavailability of such mutants in real life (Figure 7).

S130G mutation harboring cell showed resistance to the mechanism-based drug in LIVE/DEAD viability-based assay under fluorescence microscopy. We checked the residual interaction network of SDN and SDG loop in the protein, and we found less interaction of SDG residue compared with SDN. This is due to a lack of side chain in glycine against the asparagine in the SDN loop. The intramolecular residual network becomes even lesser in the S130G mutant due to replacing glycine from serine. In S130G mutants, GDG showed insufficient intra-protein residual interaction, suggesting higher dynamic and thermal instability. With all significant differences considering the effect coming from only one point mutation, our analyses provided critical insights regarding the use of combination in clinical practice. Our studies demonstrate the role of S130 residue in both structural and functional aspects. This study lays out a crucial role of serine 130 residues not only in a functional aspect (resistant against MBIs) but also in its requirement for structural stability. This issued the impossibility of *M. tuberculosis* to mutate the serine residue to become MBI resistant. This provides clinical support to use different combination therapies to treat one of the fiercest bacterial pathogens of the world.

## Data Availability Statement

The original contributions presented in the study are included in the article/Supplementary Material, further inquiries can be directed to the corresponding author/s.

## Author Contributions

SB: investigation, and writing original draft. VJ: formal analysis and methodology. NP: biophysical study and analysis. SB: biochemical study. HA: biophysical study analysis. ND: fluorescent microscopy. PR: analysis of fluorescent microscopy. AB: growth curve analysis. DG: writing review and editing. HP: editing. SH: supervision, conceptualization, methodology, software, and review and editing. All authors contributed to the article and approved the submitted version.

## Conflict of Interest

The authors declare that the research was conducted in the absence of any commercial or financial relationships that could be construed as a potential conflict of interest.

## Publisher’s Note

All claims expressed in this article are solely those of the authors and do not necessarily represent those of their affiliated organizations, or those of the publisher, the editors and the reviewers. Any product that may be evaluated in this article, or claim that may be made by its manufacturer, is not guaranteed or endorsed by the publisher.

## Footnotes

1
http://www.rcsb.org/pdb/home/home.do

## Abbreviations

AMR: antimicrobial resistance
MDR: multidrug resistant
XDR: extensively drug-resistant
MBIs: mechanism-based inhibitors
CD: circular dichroism
DSC: differential scanning calorimetry
MD: molecular dynamics
*M. tuberculosis*: *Mycobacterium tuberculosis*
TB: tuberculosis
*E. coli*: *Escherichia coli*
*K. pneumoniae*: *Klebsiella pneumonia*
*A. baumannii*: *Acinetobacter baumannii*
T_*M*_: melting temperature
His tagged: histidine tagged
WT: wild type
IPTG: isopropyl-D-1-thiogalactopyranoside
OD: optical density
CLSI: Clinical and Laboratory Standards Institute.
