# Supplementary material for: Variations in the SDN Loop of Class A Beta-Lactamases: A Study of the Molecular Mechanism of BlaC (Mycobacterium tuberculosis) to Alter the Stability and Catalytic Activity Towards Antibiotic Resistance of MBIs
Source: Front Microbiol. 2021 Oct 8;12:710291. doi: 10.3389/fmicb.2021.710291 (PMC8531524; doi:10.3389/fmicb.2021.710291)
Supplement: Supplementary file 1 [file Data_Sheet_1.docx]

Figure S1: Evolutionary analysis of beta-lactamase from different mycobacterium species. The relatedness of BlaC from *Mycobacterium tuberculosis* highlighted in red square was compared against 46 beta-lactamases from different mycobacterium species.

Figure S2: pH denaturation of wild type BlaC. Respective spectra suggest a wavelength scan from 190 to 260 nm at various pH. Elipticity value at 222nm of each of the spectra were plotted against variable pH to understand helical parameter under the influence of pH.

Figure S3: pH denaturation of S130G_BlaC. Respective spectra suggest a wavelength scan from 190 to 260 nm at various pH. Elipticity value at 222nm of each of the spectra were plotted against variable pH to understand helical parameter under the influence of pH.

Figure S4: pH denaturation of S130A_BlaC. Respective spectra suggest a wavelength scan from 190 to 260 nm at various pH. Elipticity value at 222nm of each of the spectra were plotted against variable pH to understand helical parameter under the influence of pH.

Figure S5: DSC curve of Wild type BlaC, BlaC_S130A, and BlaC_S130G

Figure S6: Interaction analysis of Sulbactam towards wild type BlaC and its variants S130A and S130G. ITC thermogram shows binding affinity of Sulbactam towards each enzyme. The upper part of each panel represent thermal power vs. time after baseline correction, bottom part represent normalized heat vs. molar ratio of reactant molecule.

| 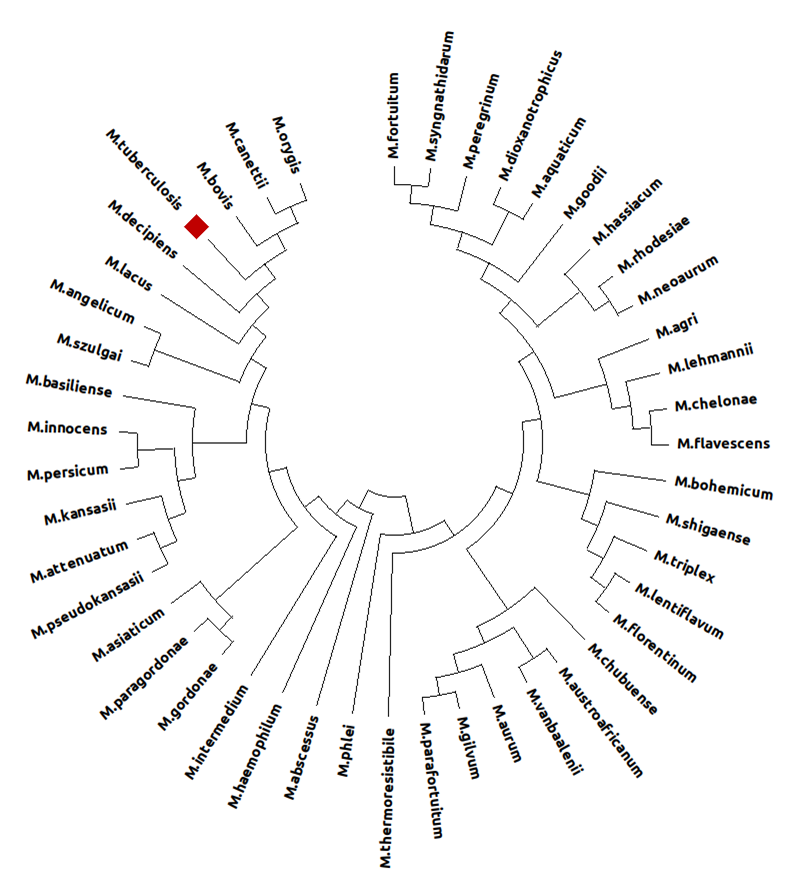 |
| --- |
| **Figure S1:** |

| 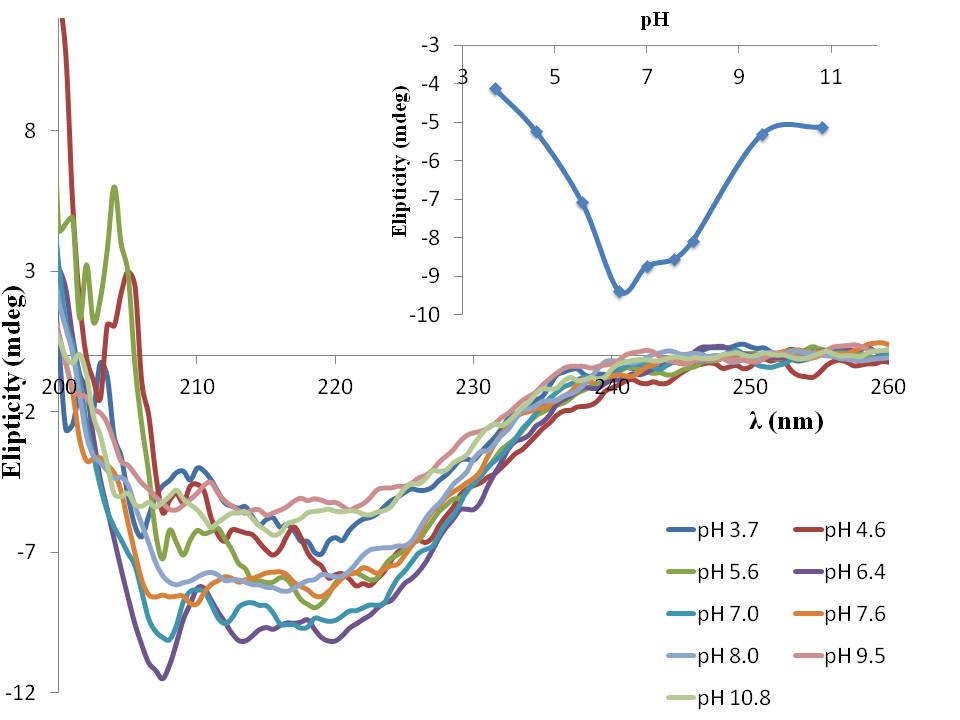 |
| --- |
| **Figure S2:** |

| 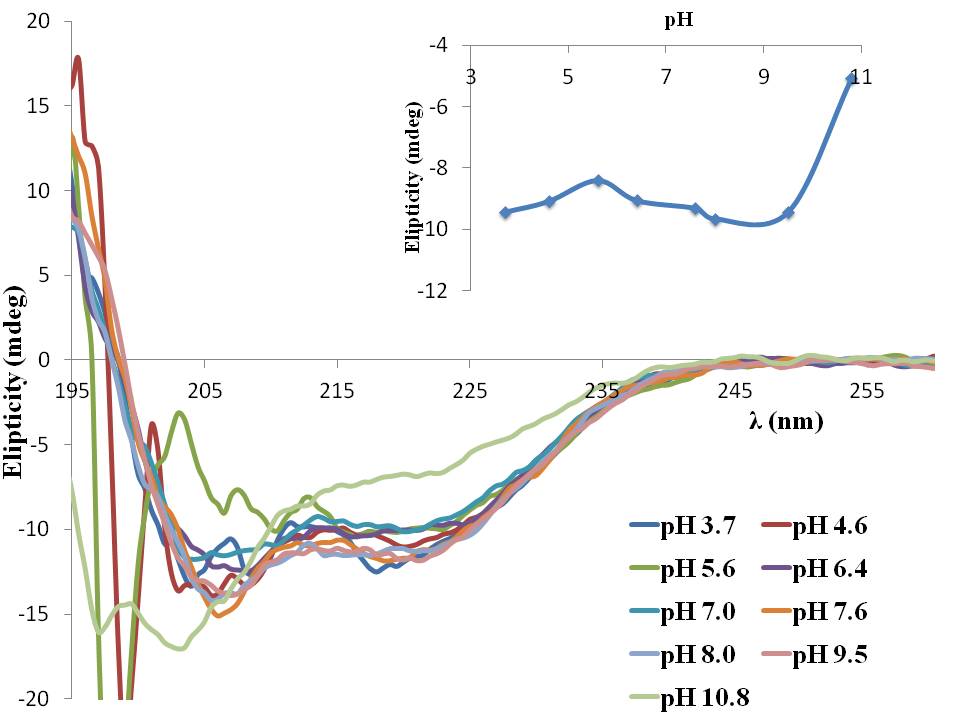 |
| --- |
| **Figure S3:** |

| 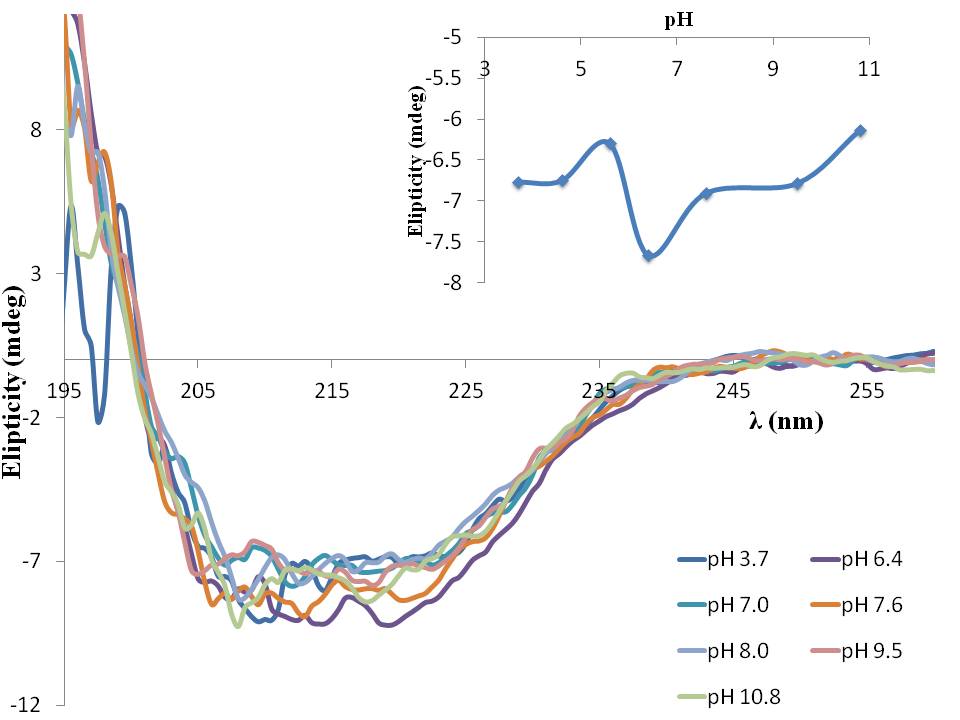 |
| --- |
| **Figure S4:** |

| 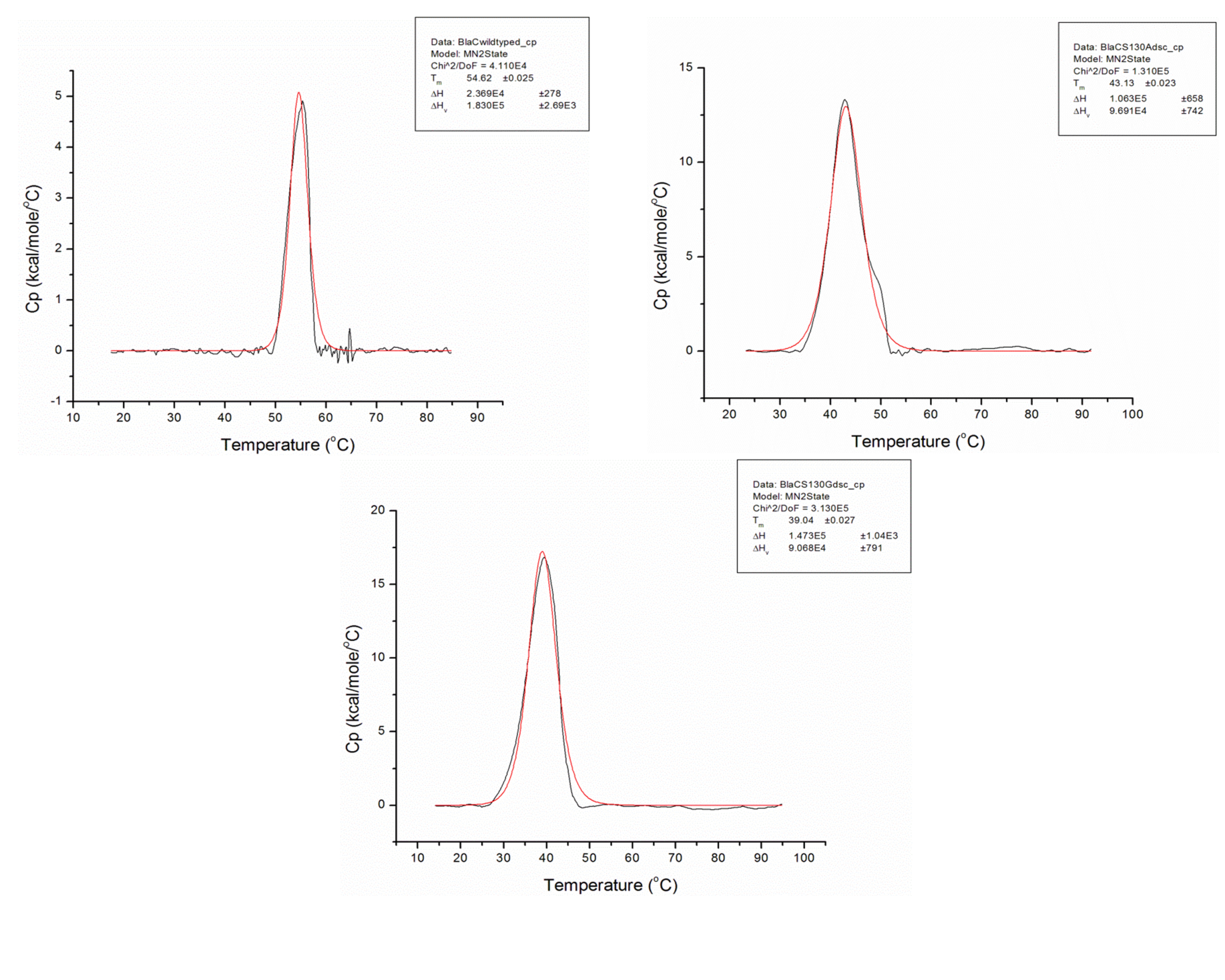 |
| --- |
| Figure S5: |

| 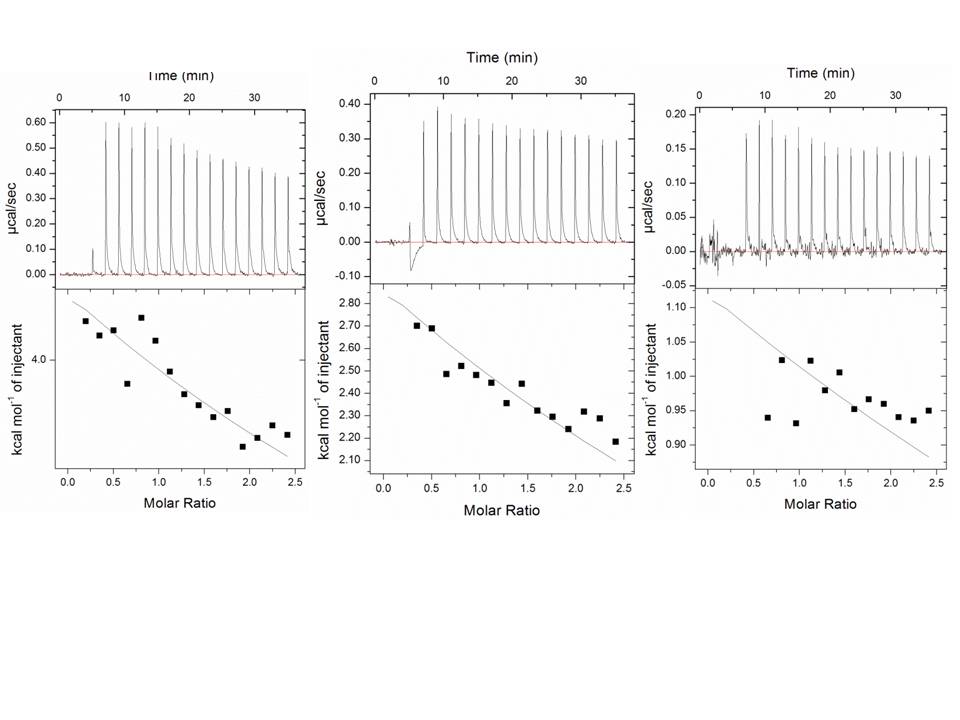 |
| --- |
| **Figure S6:** |

| **Table S1: Thermodynamic parameter obtained from isothermal titration calorimetry between wild type BlaC and its variant against Sulbactam** | | | | |
| --- | --- | --- | --- | --- |
| **Protein**  **vs** | **N** | **K_D_ (M^-1^)** | **ΔH**  **(cal/mol)** | **ΔS**  **(cal/mol/deg)** |
| Wild type BlaC+Sulbactam | 1.00 | 532 | 1.73E5 | 593 |
| S130A+Sulbactam | 1.00 | 395 | 1.46E5 | 504 |
| S130G+Sulbactam | 1.00 | 16.9 | 1.31E6 | 4.42E3 |

| **Table S2: Molecular docking analysis of Clavulanate with wild type BlaC and its variant S130G** | | | | | | |
| --- | --- | --- | --- | --- | --- | --- |
| Clavulanate | | Wt BlaC | | | Distance | 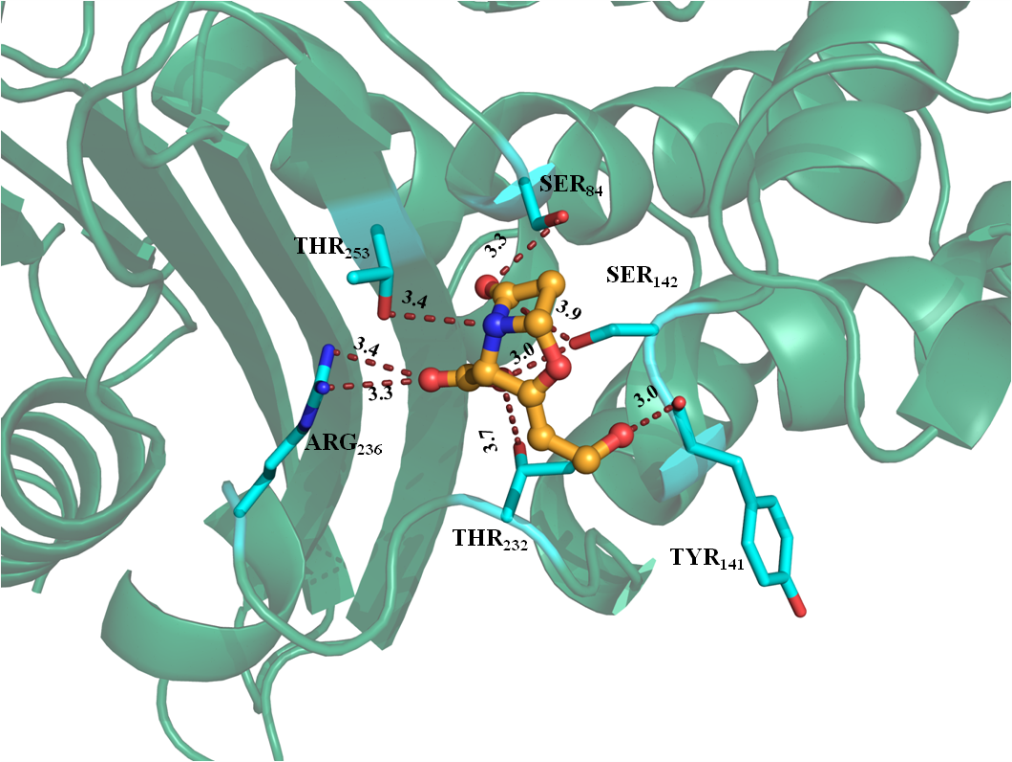  **Binding energy: -6.5 kcal/mol** |
| Clav | O10 | SER | 84 | OG | 3.3 |  |
| Clav | N1 | THR | 253 | OG1 | 3.4 |  |
| Clav | O12 | SER | 142 | OG | 3 |  |
| Clav | O10 | SER | 142 | OG | 3.9 |  |
| Clav | O12 | THR | 232 | OG1 | 3.7 |  |
| Clav | O13 | ARG | 236 | NH2 | 3.3 |  |
| Clav | O13 | ARG | 236 | NH1 | 3.4 |  |
| Clav | O14 | TYR | 141 | O | 3 |  |
| Sulbactam | | BlaC_GDG | | | Distance | 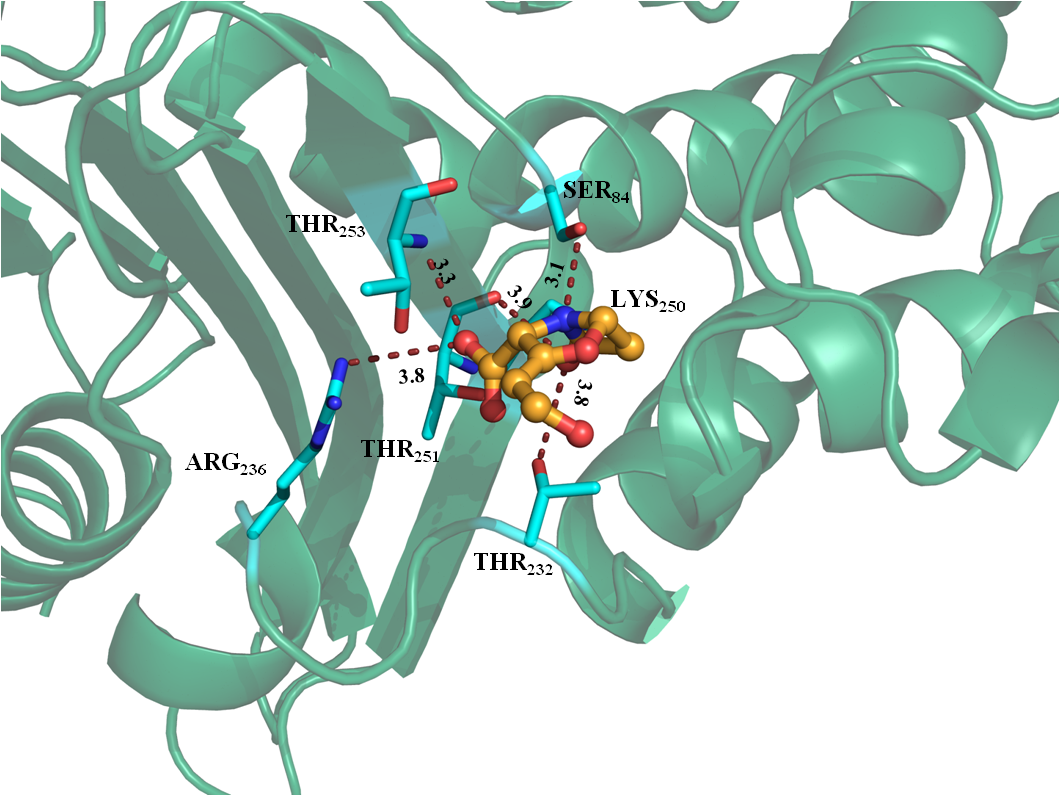  **Binding energy: -4.8 kcal/mol** |
| Sul | N1 | SER | 84 | OG | 3.09 |  |
| Sul | O10 | THR | 232 | OG1 | 3.8 |  |
| Sul | O10 | THR | 251 | O | 3.91 |  |
| Sul | O10 | LYS | 250 | NZ | 3.43 |  |
| Sul | O12 | ARG | 236 | NH1 | 3.75 |  |
| Sul | O12 | THR | 253 | N | 3.27 |  |
