# Supplementary material for: Variations in the SDN Loop of Class A Beta-Lactamases: A Study of the Molecular Mechanism of BlaC (Mycobacterium tuberculosis) to Alter the Stability and Catalytic Activity Towards Antibiotic Resistance of MBIs
Source: Front Microbiol. 2021 Oct 8;12:710291. doi: 10.3389/fmicb.2021.710291 (PMC8531524; doi:10.3389/fmicb.2021.710291)
Supplement: Supplementary file 2 [file Data_Sheet_2.docx]

| **Sequence Header used** | **GenPept ID** |
| --- | --- |
| Acidaminococcus fermentans\|ACI-1\|hlabCAbltm_1 | CAB51471.1 |
| Aeromonas_hydrophila\|AER-1\|hlabCAbltm_2 | AAC09015.1 |
| Aliivibrio_fischeri\|AFA-1\|hlabCAbltm_3 | AAR97884.1 |
| Actinomadura_sp_R39\|AMA-1\|hlabCAbltm_4 | CAA37699.1 |
| Staphylococcus_arlettae\|ARL-1\|hlabCAbltm_5 | APY23733.1 |
| Nocardia_asteroides\|AST-1\|hlabCAbltm_6 | AAG44836.1 |
| Bacillus_cereus\|BcI-1\|hlabCAbltm_7 | CAA29819.1 |
| Bacillus_anthracis\|BcI-2\|hlabCAbltm_8 | AAK53749.2 |
| Bacillus_thuringiensis\|BcI-4\|hlabCAbltm_9 | CAA58448.1 |
| Bacillus_mycoides\|BcI-5\|hlabCAbltm_10 | CAA44161.1 |
| Bacillus_clausii\|BCL-1\|hlabCAbltm_11 | ALA52629.1 |
| Klebsiella_pneumoniae\|BEL-1\|hlabCAbltm_12 | AHD24679.1 |
| Pseudomonas_aeruginosa\|BEL-2\|hlabCAbltm_13 | ACV69996.1 |
| Serratia_marcescens\|BES-1\|hlabCAbltm_14 | AAF61147.1 |
| Pseudomonas_fluorescens\|BIC-1\|hlabCAbltm_15 | ADB56658.1 |
| Mycobacterium_tuberculosis\|BlaC\|hlabCAbltm_16 | AMQ38896.1 |
| Mycobacterium_smegmatis\|BlaS\|hlabCAbltm_17 | AIU14458.1 |
| Bordetella_bronchiseptica_RB50\|BOR-1\|hlabCAbltm_18 | CAE32545.1 |
| Burkholderia_pseudomallei\|BPS-1\|hlabCAbltm_19 | AAK37506.1 |
| Burkholderia_thailandensis_2002721643\|BPS-3\|hlabCAbltm_20 | AJY01305.1 |
| Burkholderia_sp_2002721687\|BPS-4\|hlabCAbltm_21 | AJY38930.1 |
| Burkholderia_oklahomensis\|BPS-5\|hlabCAbltm_22 | AIO70431.1 |
| Moraxella_catarrhalis\|BRO-1\|hlabCAbltm_23 | AAA92126.1 |
| Carnobacterium_divergens\|CAD-1\|hlabCAbltm_24 | AAV65950.1 |
| Achromobacter_xylosoxidans\|CARB-2\|hlabCAbltm_25 | ANI26091.1 |
| Acinetobacter_baumannii\|CARB-4\|hlabCAbltm_26 | ACN89662.1 |
| Acinetobacter_calcoaceticus_subsp_anitratus\|CARB-5\|hlabCAbltm_27 | AAF61417.1 |
| Vibrio_cholerae\|CARB-6\|hlabCAbltm_28 | AAD19217.1 |
| Oligella_urethralis\|CARB-8\|hlabCAbltm_29 | AAO59455.1 |
| Proteus_mirabilis\|CARB-12\|hlabCAbltm_30 | BAA02497.1 |
| Vibrio_parahaemolyticus\|CARB-17\|hlabCAbltm_31 | AIL92326.1 |
| Vibrio_alginolyticus\|CARB-42\|hlabCAbltm_32 | ALR93825.1 |
| Salmonella_enterica\|CARB-55\|hlabCAbltm_33 | WP_071846358.1 |
| Bacteroides_sp_4_1_36\|CblA\|hlabCAbltm_34 | EFV25597.1 |
| Clostridium_botulinum\|CBP-1\|hlabCAbltm_35 | ALF06101.1 |
| Citrobacter_koseri\|CdiA\|hlabCAbltm_36 | CAA44485.1 |
| Bacteroides_fragilis\|CepA\|hlabCAbltm_37 | AAA21532.1 |
| Bacteroides_cellulosilyticus\|CepA-6\|hlabCAbltm_38 | KXU41062.1 |
| Bacteroidales_bacterium_KA00251\|CfxA-2\|hlabCAbltm_39 | KXB36938.1 |
| Bacteroides_caccae\|CfxA-3\|hlabCAbltm_40 | CUQ50797.1 |
| Bacteroides_vulgatus_str._3775_SL(B)_10_(iv)\|CfxA-5\|hlabCAbltm_41 | KDS26384.1 |
| Prevotella_intermedia\|CfxA-6\|hlabCAbltm_42 | CAY19194.1 |
| uncultured_organism\|CfxA-7\|hlabCAbltm_43 | ACT97371.1 |
| Prevotella_bivia\|CfxA-8\|hlabCAbltm_44 | AAM48116.1 |
| Prevotella_melaninogenica\|CfxA-10\|hlabCAbltm_45 | AAM48124.1 |
| Prevotella_oralis\|CfxA-11\|hlabCAbltm_46 | AAM48125.1 |
| Prevotella_buccae\|CfxA-12\|hlabCAbltm_47 | AAM48117.1 |
| Prevotella_denticola\|CfxA-13\|hlabCAbltm_48 | AAM48121.1 |
| Chryseobacterium_gleum\|CGA-1\|hlabCAbltm_49 | AAL55262.1 |
| Chryseobacterium_indologenes\|CIA-1\|hlabCAbltm_50 | BAL40892.1 |
| Elizabethkingia_anophelis\|CME-1\|hlabCAbltm_51 | KUY21918.1 |
| Elizabethkingia_meningoseptica\|CME-2\|hlabCAbltm_52 | AAF19261.1 |
| Chromobacterium_piscinae\|CRP-1\|hlabCAbltm_53 | KIA78998.1 |
| Chromobacterium_sp_C-61\|CRS-1\|hlabCAbltm_54 | WP_039755574.1 |
| Chromobacterium_haemolyticum\|CRS-2\|hlabCAbltm_55 | WP_043592266.1 |
| Capnocytophaga_sputigena\|CSP-1\|hlabCAbltm_56 | ADD82978.1 |
| Escherichia_coli\|CTX-M-1\|hlabCAbltm_57 | ABK06383.1 |
| Kluyvera_ascorbata\|CTX-M-5\|hlabCAbltm_58 | CAB63259.1 |
| Citrobacter_amalonaticus\|CTX-M-8\|hlabCAbltm_59 | AAF04388.1 |
| Enterobacter_cloacae\|CTX-M-9\|hlabCAbltm_60 | AAZ30046.1 |
| Enterobacter_hormaechei_subsp_oharae\|CTX-M-12\|hlabCAbltm_61 | KJL64335.1 |
| Citrobacter_freundii\|CTX-M-14\|hlabCAbltm_62 | ABN64105.1 |
| Klebsiella_sp_ARS06-441\|CTX-M-68\|hlabCAbltm_63 | ABV81082.1 |
| Providencia_stuartii\|CTX-M-75\|hlabCAbltm_65 | ACS32294.1 |
| Kluyvera_georgiana\|CTX-M-78\|hlabCAbltm_66 | CAQ42481.2 |
| Shigella_sp_SH219\|CTX-M-107\|hlabCAbltm_67 | AEM44650.1 |
| Shigella_sp_SH223\|CTX-M-108\|hlabCAbltm_68 | AEM44651.1 |
| Shigella_sp_SH361\|CTX-M-109\|hlabCAbltm_69 | AEM44654.1 |
| Shigella_sp_SH165\|CTX-M-110\|hlabCAbltm_70 | AEM44648.1 |
| Shigella_sp_SH202\|CTX-M-111\|hlabCAbltm_71 | AEM44649.1 |
| Shigella_sp_SH257\|CTX-M-112\|hlabCAbltm_72 | AEM44652.1 |
| Shigella_sp_SH284\|CTX-M-113\|hlabCAbltm_73 | AEM44653.1 |
| Providencia_rettgeri\|CTX-M-114\|hlabCAbltm_74 | ACU00153.1 |
| Kluyvera_sp_MRB7\|CTX-M-152\|hlabCAbltm_75 | AHY20039.1 |
| Klebsiella_oxytoca\|CTX-M-162\|hlabCAbltm_76 | AKO63213.1 |
| uncultured_bacterium\|CTX-M-206\|hlabCAbltm_77 | AIC64344.1 |
| Proteus_vulgaris\|CumA-1\|hlabCAbltm_78 | CAA56427.1 |
| Desulfovibrio_desulfuricans\|DES-1\|hlabCAbltm_80 | AAM45855.1 |
| Nocardia_farcinica\|FAR-1\|hlabCAbltm_81 | WP_070064531.1 |
| Serratia_fonticola\|FONA-1\|hlabCAbltm_82 | AKG71438.1 |
| Enterobacter_asburiae\|FRI-2\|hlabCAbltm_84 | ANZ90381.1 |
| Francisella_tularensis\|FTU-1\|hlabCAbltm_85 | KFJ38081.1 |
| Enterobacter_hormaechei_subsp_steigerwaltii\|GES-2\|hlabCAbltm_86 | KJX27876.1 |
| Citrobacter_gillenii\|GIL-1\|hlabCAbltm_87 | ABL74511.1 |
| Escherichia_hermannii\|HER-1\|hlabCAbltm_88 | AAL26797.1 |
| Proteus_penneri\|HugA\|hlabCAbltm_89 | AAL57765.1 |
| Enterobacter_sp_50588862\|IMI-9\|hlabCAbltm_90 | KSX64667.1 |
| Enterobacter_hormaechei\|IMI-14\|hlabCAbltm_91 | APY16311.1 |
| Kluyvera_intermedia\|KLUA-12\|hlabCAbltm_92 | AAV93320.1 |
| Kluyvera_cryocrescens\|KLUC-1\|hlabCAbltm_93 | AAK08976.1 |
| Kluyvera_sp_N03-0461\|KLUY-2\|hlabCAbltm_94 | AAT45026.1 |
| Kluyvera_sp_N03-0460\|KLUY-3\|hlabCAbltm_95 | AAT45022.1 |
| Kluyvera_sp_N03-0462\|KLUY-4\|hlabCAbltm_96 | AAT45018.1 |
| Citrobacter_sp_MGH_55\|KPC-4\|hlabCAbltm_97 | KDF02556.1 |
| Klebsiella_variicola\|LEN-7\|hlabCAbltm_98 | CTQ11867.1 |
| Klebsiella_sp_1_1_55\|LEN-27\|hlabCAbltm_99 | WP_032700972.1 |
| Klebsiella_sp_KTE92\|LEN-31\|hlabCAbltm_100 | EOQ54266.1 |
| Pedobacter_lusitanus\|LUS-1\|hlabCAbltm_101 | KIO77103.1 |
| Mycobacterium_fortuitum\|MFO-1\|hlabCAbltm_102 | AAA19882.1 |
| Janthinobacterium_sp_Marseille\|MIN-1\|hlabCAbltm_103 | ABR90152.1 |
| Oceanobacillus_iheyensis_HTE831\|OIH-1\|hlabCAbltm_105 | BAC14749.1 |
| Raoultella_ornithinolytica\|ORN-2\|hlabCAbltm_106 | AAS87024.1 |
| Klebsiella_michiganensis\|OXY-1-1\|hlabCAbltm_107 | AEX04382.1 |
| Enterococcus_faecalis_CH188\|PC1-1\|hlabCAbltm_108 | EEU86309.1 |
| Staphylococcus_aureus\|PC1-2\|hlabCAbltm_109 | AAA26647.1 |
| Staphylococcus_argenteus\|PC1-5\|hlabCAbltm_110 | CDR25766.1 |
| Enterococcus_faecalis\|PC1-8\|hlabCAbltm_111 | AAB40888.1 |
| Staphylococcus_intermedius\|PC1-19\|hlabCAbltm_114 | ABK96857.1 |
| Streptococcus_pneumoniae\|PC1-45\|hlabCAbltm_115 | CRG02023.1 |
| Staphylococcus_capitis\|PC1-47\|hlabCAbltm_116 | CUT96129.1 |
| Staphylococcus_warneri\|PC1-74\|hlabCAbltm_117 | WP_049401912.1 |
| Staphylococcus_equorum\|PC1-120\|hlabCAbltm_118 | OEL07569.1 |
| Staphylococcus_sp_HMSC073C02\|PC1-121\|hlabCAbltm_119 | OFJ59084.1 |
| Staphylococcus_sp_HMSC58B01\|PC1-122\|hlabCAbltm_120 | OFO18104.1 |
| Staphylococcus_sp_HMSC58E11\|PC1-123\|hlabCAbltm_121 | OHS34358.1 |
| Staphylococcus_sp_HMSC74G01\|PC1-124\|hlabCAbltm_122 | OHS78034.1 |
| Staphylococcus_simulans_bv._staphylolyticus\|PC1-141\|hlabCAbltm_123 | YP_003505728.1 |
| Paenibacillus_polymyxa\|PC1-155\|hlabCAbltm_124 | WP_038978284.1 |
| Paramesorhizobium_deserti\|PAD-1\|hlabCAbltm_125 | KXF74838.1 |
| Staphylococcus_xylosus\|PC2-2\|hlabCAbltm_126 | CCM44120.1 |
| Burkholderia_cepacia\|PenA\|hlabCAbltm_127 | AAB53622.1 |
| Burkholderia_cenocepacia\|PenB-1\|hlabCAbltm_128 | ACJ63454.1 |
| Bacillus_licheniformis\|PenP-1\|hlabCAbltm_130 | CAA23431.1 |
| Raoultella_planticola_ATCC_33531\|PLA-1\|hlabCAbltm_131 | AAP79046.1 |
| Raoultella_planticola\|PLA-2a\|hlabCAbltm_132 | AAP79049.1 |
| Pseudovibrio_sp_1D03\|PSV-1\|hlabCAbltm_133 | API65130.1 |
| Rahnella_aquatilis\|RAHN-1\|hlabCAbltm_134 | AAK83220.1 |
| Rahnella_sp_42-Carrot\|RAHN-2\|hlabCAbltm_135 | ADO51656.1 |
| Rhodobacter_capsulatus\|RCAP-1\|hlabCAbltm_136 | CAA33795.1 |
| Actinobacillus_pleuropneumoniae\|ROB-1\|hlabCAbltm_137 | AAB24384.1 |
| Serratia_rubidaea\|RUB-1\|hlabCAbltm_138 | ANV82483.1 |
| Streptomyces_albus\|SDA-A-1\|hlabCAbltm_139 | AAA26775.1 |
| Streptomyces_cacaoi\|SDA-D-1\|hlabCAbltm_140 | BAA14224.1 |
| Streptomyces_sp_NRRL_S-1868\|SDA-D-2\|hlabCAbltm_141 | WP_030877515.1 |
| Streptomyces_sp_NRRL_F-5053\|SDA-D-3\|hlabCAbltm_142 | WP_030892446.1 |
| Streptomyces_cellulosae\|SDA-E-1\|hlabCAbltm_143 | BAA02176.1 |
| Streptomyces_fradiae\|SDA-F-1\|hlabCAbltm_144 | AAA26709.1 |
| Streptomyces_clavuligerus_ATCC_27064\|SDA-G-1\|hlabCAbltm_145 | CAA90895.1 |
| Streptomyces_lavendulae\|SDA-L-1\|hlabCAbltm_146 | AAA26708.1 |
| Citrobacter_sedlakii\|SED-1\|hlabCAbltm_147 | AAK63223.1 |
| Sphingobium_japonicum_UT26S\|SGM-2\|hlabCAbltm_148 | BAI97006.1 |
| Sphingobium_indicum_B90A\|SGM-3\|hlabCAbltm_149 | EIM65997.1 |
| Sphingobium_chlorophenolicum_L-1\|SGM-4\|hlabCAbltm_150 | AEG48329.1 |
| Sphingobium_yanoikuyae_ATCC_51230\|SGM-6\|hlabCAbltm_151 | EKU76984.1 |
| Sphingobium_yanoikuyae\|SGM-7\|hlabCAbltm_152 | WP_010337133.1 |
| Enterobacter_sp_MGH_14\|SHV-7\|hlabCAbltm_153 | ERP00116.1 |
| Ewingella_sp_SMO-1\|SMO-1\|hlabCAbltm_154 | AGM37757.1 |
| Capnocytophaga_ochracea\|TEM-17\|hlabCAbltm_156 | CAA74912.2 |
| Enterobacter_aerogenes_EA1509E\|TEM-24\|hlabCAbltm_157 | CCG28759.1 |
| Enterobacter_sp_GN02186\|TEM-26\|hlabCAbltm_158 | KLG19745.1 |
| Shigella_sonnei\|TEM-32\|hlabCAbltm_159 | CTA52364.1 |
| Morganella_morganii\|TEM-72\|hlabCAbltm_160 | AAF19151.1 |
| Enterobacter_aerogenes\|TEM-114\|hlabCAbltm_162 | AAS89984.1 |
| Enterobacter_sp_SP040079\|TEM-150\|hlabCAbltm_164 | ACI32333.1 |
| Acinetobacter_haemolyticus\|TEM-162\|hlabCAbltm_165 | ABO64442.1 |
| Haemophilus_parainfluenzae\|TEM-182\|hlabCAbltm_168 | ADP20705.1 |
| Neisseria_gonorrhoeae\|TEM-220\|hlabCAbltm_169 | AIW68620.1 |
| Acinetobacter_pittii\|VEB-3\|hlabCAbltm_170 | ACX94071.1 |
| Vibrio_harveyi\|VDA-H-1\|hlabCAbltm_171 | AAF23817.1 |
| Yersinia_enterocolitica\|YENT-1\|hlabCAbltm_172 | CAA40357.1 |
